# An investigation and validation of CT scan in detection of spinal epidural adipose tissue

**DOI:** 10.1097/MD.0000000000019448

**Published:** 2020-03-06

**Authors:** Yilei Chen, Ziang Hu, Zhaozhi Li, Shunwu Fan, Xing Zhao, Lijiang Song, Lili Wang

**Affiliations:** a Department of Orthopaedics Surgery, Sir Run Run Shaw Hospital, School of Medicine, Zhejiang University; b School of Statistics and Mathematics, Zhejiang Gongshang University, Hangzhou, PR China.

**Keywords:** computed tomography, epidural fat, lumbar epidural lipomatosis, magnetic resonance imaging

## Abstract

To investigate the accuracy of computed tomography (CT) in evaluating spinal epidural adipose tissue compared to magnetic resonance imaging (MRI).

CT scan images and matched magnetic resonance images of total 368 patients between July 2014 and July 2016 were evaluated. Hounsfield units (HU) of epidural fat (EF), dural sac (DuS), ligamentum flavum, bone of facet joints, and paraspinal muscles were measured for comparison. Anteroposterior diameter of the EF, anteroposterior diameter of the DuS, transverse diameter of the DuS, cross-sectional area of the EF, and cross-sectional area of the DuS were measured at each disc level from L1–2 to L5–S1.

Fat tissue showed exclusive negative HU significantly different from all other periphery tissues. Pearson correlation coefficient analyses showed significant positive correlations between CT and MRI measurements; Bland–Altman plots also depicted satisfied agreement. Overgrowth of spinal EF was more commonly found at L2–3 and L3–4 levels in present study, and body weight, age, and gender were significantly associated with amounts of EF both on CT and MRI.

The CT scan is a satisfied alternative of MRI for the evaluation of spinal epidural adipose tissue.

## 1 Introduction

Lumbar spinal stenosis is a narrowing of spaces in the central lumbar spinal canal, lateral recess, or foramen that produces pressure on the spinal cord and/or nerve roots, which further causes back pain, radicular pain, or numbness to the legs, and claudication.^[1]^ The vast majority of lumbar spinal stenosis results from degenerative changes including: disc herniation, hypertrophy of the facet joints and the ligamentum flavum, osteophytosis, and spondylolisthesis.^[2]^ Recently, an uncommon cause of spinal canal stenosis was reported and investigated, which is, namely, spinal epidural lipomatosis (SEL),^[3,4]^ or more specifically, lumbar epidural lipomatosis (LEL).^[5–7]^ LEL is characterized by an excessive deposit of normal unencapsulated adipose tissue in the epidural space causing compression of the nerve roots and the spinal cord.^[8]^ Although several related factors like hypercortisolism, obesity, hypothyroidism, hyperprolactinemia, and protease inhibitors in high-intensity antiretroviral therapy have been reported, the underlying pathological mechanism is still unknown.^[9]^ The excessive epidural fat (EF) usually occurs in the spinal epidural space of thoracic and lumbar spine region.^[10]^ Patients with symptomatic LEL usually present with localized, chronic back pain similar to those in spinal canal stenosis.^[11]^

Diagnosis of LEL requires clinical suspicion, imaging studies, and surgical evaluation as well. Magnetic resonance imaging (MRI) is considered as the most sensitive and specific tool to assess epidural fatty tissue.^[7]^ However, its relatively high financial cost, long duration of scanning, and certain contraindications such as claustrophobia, limited its application. Meanwhile, previous researches have used high solution of computed tomography (CT) scanning as an effective and specific tool in fat detection: Lovejoy et al^[12]^ used abdominal CT scan to assess the relationships between visceral fat and insulin sensitivity index. Harley and Pickford ^[13]^ used CT scan to assess the thickness of the subcutaneous fat layer at the level of the umbilicus and the suprapubic region. Moreover, Dahlen et al^[14]^ used CT scan to assess intravestibular lipoma. However, the accuracy, effectiveness, and specificity of CT scan in detection of adipose tissue in spinal canal is still lacking in literature. In the present study, we investigated the ability of CT scan in detection of spinal epidural adipose tissue, and its effectiveness and specificity compared to MRI.

## 2 Materials and methods

### 2.1 Patients

From July 2014 to July 2016, all patients admitted to our clinic that accepted both CT scan and MRI of lumbar spine were included. Images of this study were extracted from our database without unnecessary patient information, no patient consent or ethics review is needed. The exclusion criteria were as followed: spondylitis and discitis with extradural abscess, extradural tumors, lumbosacral trauma or pathologic fractures (e.g., metastasis) with anatomic distortion of the spinal canal, previous lumbosacral surgery or radiotherapy, and severe posterior disc extrusion located between the entrance zones of the neural foramina with notable ventral thecal sac compression. Of 531 patients identified, 368 (195 men and 173 women; age range 18–86 years) with totally 1221 disc levels (L1–2 n = 192, L2–3 n = 237, L3–4 n = 360, L4–5 n = 320, and L5–S1 n = 112) were included in this analysis. Sociodemographic characteristics, including age, gender, body height, body weight, and body mass index (BMI) were recorded (Table 1).

**Table 1:**
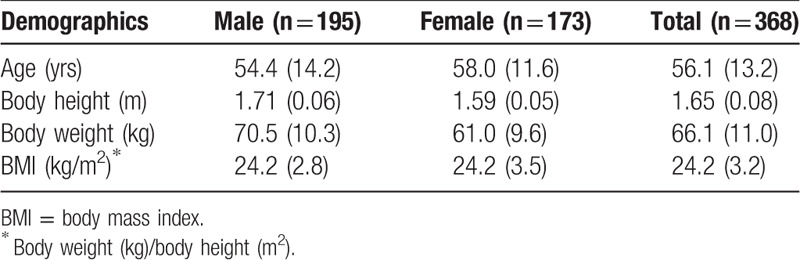
Demographics of the study population.

### 2.2 Imaging parameters

Both CT and MRI examinations were performed with patients lying in supine position. CT scans were performed on a Siemens (CT: Siemens, Forchheim, Germany) SOMATOM Sensation 16-slice CT scanner, using a standard sequential scan protocol (120 KV, 140 mAs, 4-mm slice thickness and 1 mm interslice gap). MRI examinations were performed on a General Electric (MRI: GE Healthcare, Milwaukee, Wisconsin) 1.5-T magnet. Sequences includes as following: Spin-echo (SE) T1-weighted sagittal images (TR 560 ms/TE 12 ms, with a 320 × 256 matrix, 3 excitations, 4-mm slice thickness, and 1 mm slice gap); SE T2-weighted axial images (TR 3000 ms/TE100 ms, with a 320 × 56 matrix, 3 excitations, 4-mm slice thickness, and 1 mm slice gap). CT scan and magnetic resonance (MR) image were performed within no more than 4 weeks to minimize any possible fat tissue proliferation or regression.

### 2.3 Measurements

CT windows were optimized as: level 40 Hounsfield units (HU) and width 350 HU for HU value measurements, or level 55 HU and width 255 HU for morphology parameter measurements; T2-weighted axial MRI images were also obtained for analysis. To establish reproducible measurements of dural sac (DuS) and EF for each level, images were analyzed on the axial plane parallel and tangent to the superior end plate of the lower vertebral body.

**Figure 1:**
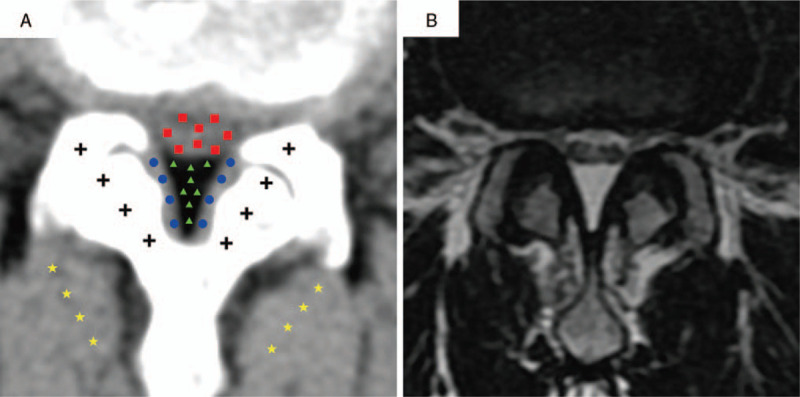
Illustration of HU value measurements. Axial CT image of intervertebral disc plane and axial T2WI MRI image corresponding CT. HU values of epidural fat (triangle), dural sac (square), ligamentum (circle), bone of the facet joints (cross), and paraspinal muscles (pentagram) were measured at designed 8 dots separately.

**Figure 2:**
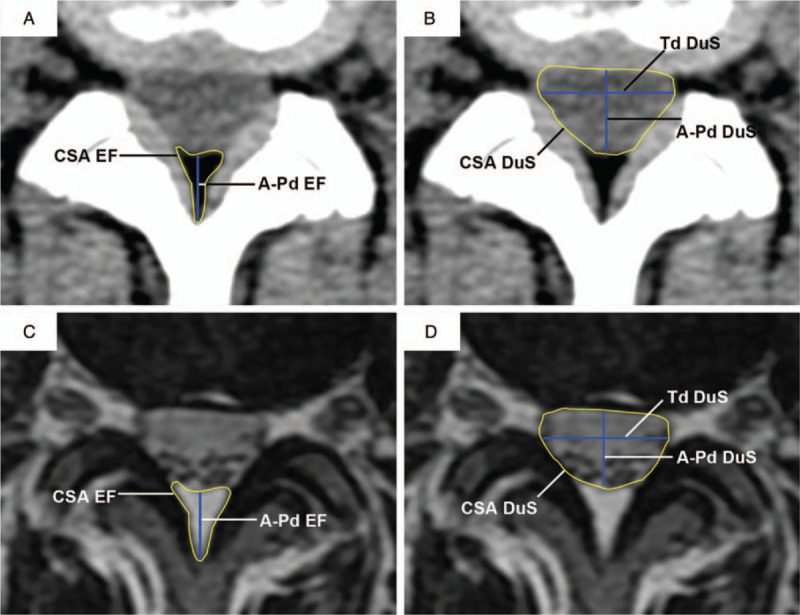
Illustration of diameter and cross-sectional area measurements. (A and C) Epidural fat measurements on axial CT and matched axial T2WI MR images. (B and D) Dural sac measurements on axial CT and matched axial T2WI MR images. A-Pd EF = anteroposterior diameter of the epidural fat, A-Pd DuS = anteroposterior diameter of the dural sac, CT = computed tomography, MR = magnetic resonance.

For each intervertebral interval, HU values of EF (triangle) and periphery tissues including DuS (square), ligamentum flavum (circle), bone of facet joints (cross), and paraspinal muscles (pentagram) were assessed at designed 8 dots as showed in Figure 1. Results were represented as mean ± SD of HU values. Furthermore, 3 linear and 2 area measurements that represent parameters of spinal canal at intervertebral disc plane were obtained from both CT and MRI images, respectively, as showed in Figure 2:

1. Anteroposterior diameter of the epidural fat (A-Pd EF),
2. Anteroposterior diameter of the dural sac (A-Pd DuS),
3. Transverse diameter of the dural sac (Td DuS),
4. Cross-sectional area of the epidural fat (CSA EF),
5. Cross-sectional area of the dural sac (CSA DuS).

The measurement of HU values in this study was performed on PACS. The measurements of morphology parameters were made with ImagJ 1.48 (National Institutes of Health, http://rsbweb.nih.gov/ij/download.html). Analysis of these images was carried out by a medical student and a senior orthopedic surgeon who were blind to patients’ identity information and radiology reports.

### 2.4 Statistical analysis

The SPSS version 11 computer statistics package (SPSS, Inc, Chicago, IL) was used to perform statistical tests. One-way ANOVA was used to determine the presence of statistically significant differences of HU values among different tissues. Pearson correlation coefficient was used to evaluate correlations between CT and MRI measurements, and the primary data were displayed on scatter plots with linear fits. Bland–Altman plots were performed to assess the agreement between the 2 methods. Paired student *t* test was used to compare diameter and area measurements between CT and MRI. Kappa test were used to compare radiological diagnosis consistency of LEL between CT and MRI. Chi-squared test was used to determine which level was most frequently affected by LEL. Finally, multiple linear regression analysis was conducted to assess relationship between EF thickness and patients’ characteristics including gender, age, body height, and body weight.

## 3 Results

### 3.1 Patient characteristics

A total of 368 patients were enrolled in this retrospective study (195 males, 173 female). Their mean age was 56.1 ± 13.2 years (range, 18–86 years), the mean body height was 1.65 ± 0.08 m (range, 1.47–1.86 m), the mean body weight was 66.1 ± 11.0 kg (range, 42.0–110.0 kg), and the mean BMI was 24.2 ± 3.2 (range, 17.4–35.8).

### 3.2 Detection of spinal EF by CT scan

**Figure 3:**
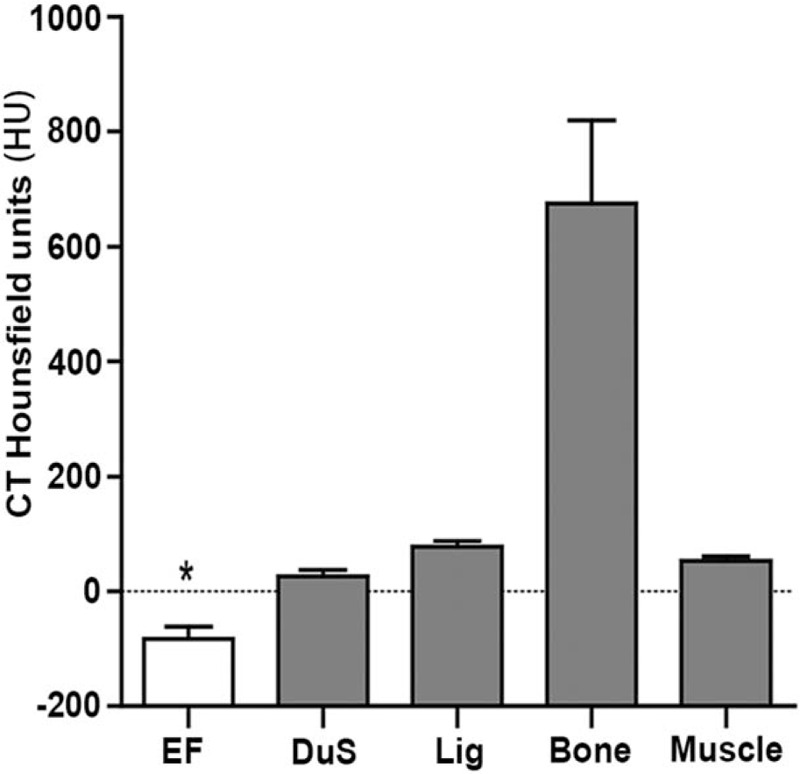
HU value comparison among epidural fat and periphery tissues. HU value of epidural fat was remarkably lower than those of periphery tissues (∗, *P* < .001), and HU values among all tissues were significantly different (all *P* < .001). Bone = bone of the facet joints, CT = computed tomography, DuS = dural sac, EF = epidural fat, HU = Hounsfield units, Lig = ligamentum flavum, Muscle = paraspinal muscles.

To assess the ability and specificity of detecting fat tissue by using CT scan, HU value of EF, DuS, ligamentum, bone, and paraspinal muscles were assessed and recorded as illustrated in Figure 1. The HU value of each tissue was as follows: EF, −82 ± 23 HU; DuS, 26 ± 12 HU; ligamentum, 78 ± 11 HU; bone, 672 ± 143 HU; and paraspinal muscles, 53 ± 9 HU. Comparison of these HU values was shown in Figure 3. HU values were significantly different among all these tissues (Fig. 3, bar graph, *P* < .001). Among these, bone tissue showed the highest average HU value with its peak reaching more than 1000 HU; by contrast, EF showed the lowest, exclusive negative HU value.

**Figure 4:**
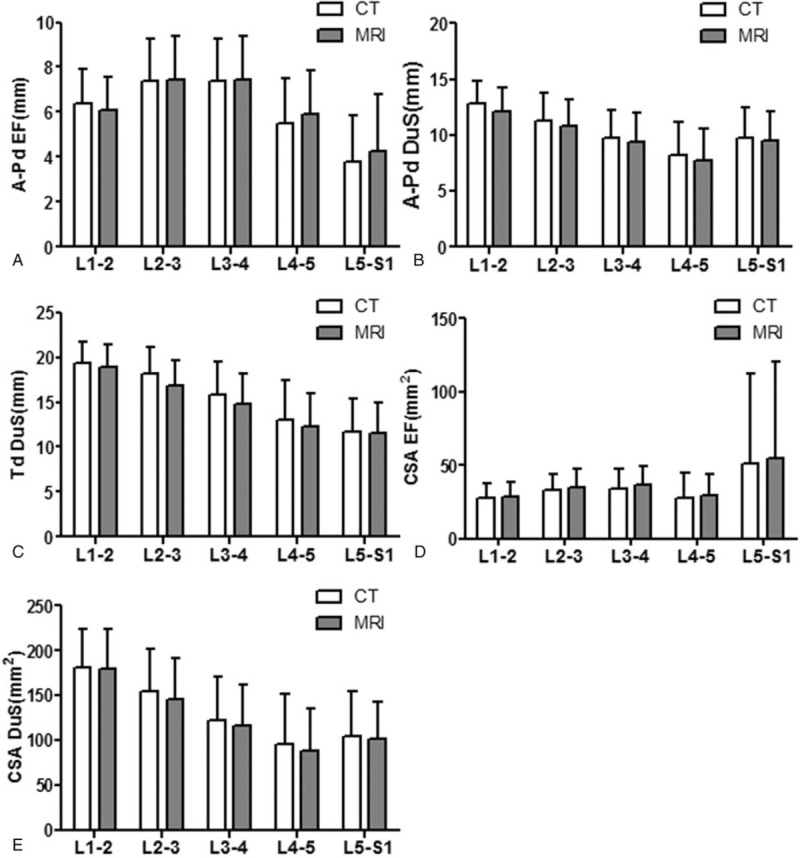
Comparison of parameters including A-Pd EF, A-Pd DuS, Td DuS, CSA EF, and CSA DuS at 5 intervertebral intervals in CT and MRI measurements. No difference was detected between CT and MRI measurements of all parameters. A-Pd EF = anteroposterior diameter of the epidural fat, A-Pd DuS = anteroposterior diameter of the dural sac, CSA DuS = cross-sectional area of the dural sac, CSA EF = cross-sectional area of the epidural fat, CT = computed tomography, MRI = magnetic resonance imaging, Td DuS = transverse diameter of the dural sac.

**Figure 5:**
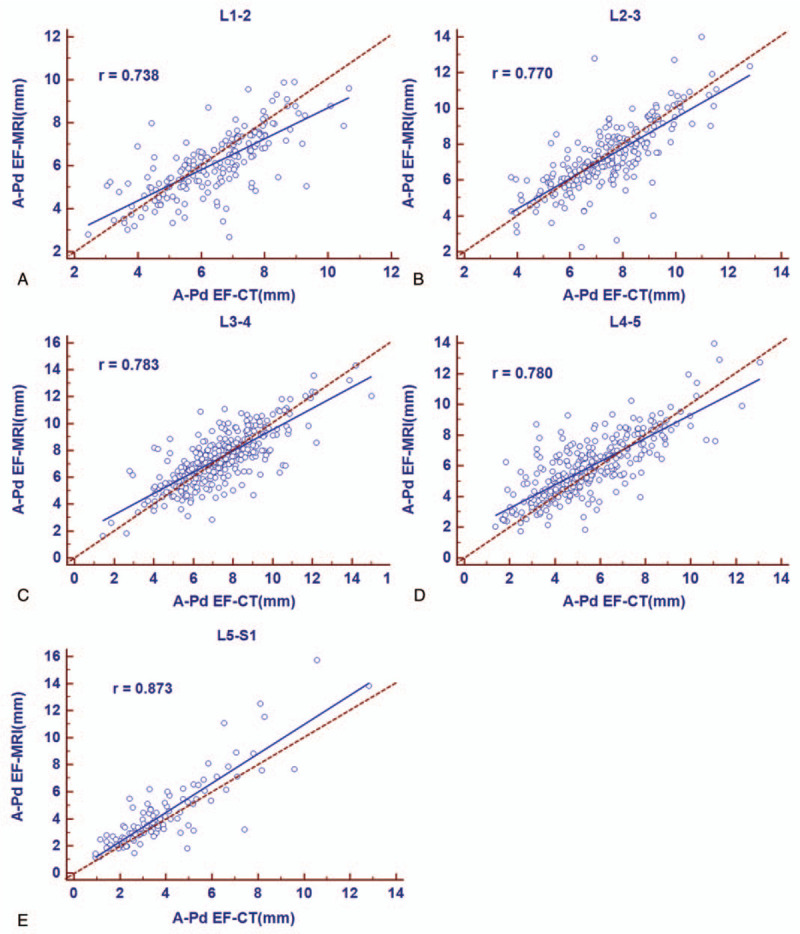
Relationship of A-Pd EF between CT and MRI at L1–2 to L5–S1. Pearson correlation analysis shows a significant positive correlation of fat diameters between CT and MRI at all 5 disc levels (L1–2 Pearson r = 0.738, *P* < .001, n = 192; L2–3 Pearson r = 0.770, *P* < .001, n = 237; L3–4 Pearson r = 0.783, *P* < .001, n = 360; L4–5 Pearson r = 0.780, *P* < .001, n = 320; L5–S1 Pearson r = 0.873, *P* < .001, n = 112). A-Pd EF = anteroposterior diameter of the epidural fat, CT = computed tomography, MRI = magnetic resonance imaging.

To further explore the accuracy of adipose tissue measurement on CT scan compared to MRI, the measurements of A-Pd EF, A-Pd DuS, Td DuS, CSA EF, and CSA DuS were obtained on both CT and MR images, as shown in Figure 2. No significant difference between matched measurement from CT and MRI was found in any tissue parameter of this study (Fig. 4), indicating a reliable assessment of fat tissue on CT scan. We next examined correlation and consistency of fat tissue measurement results between matched CT and MRI at each disc level. Significant positive correlations were observed between CT and MRI measurements of A-Pd EF at all disc levels. Pearson coefficients were 0.738, 0.770, 0.783, 0.780, and 0.873 from L1–2 to L5–S1, respectively, showed a satisfied correlation (all *P* < .001, Fig. 5).

**Figure 6:**
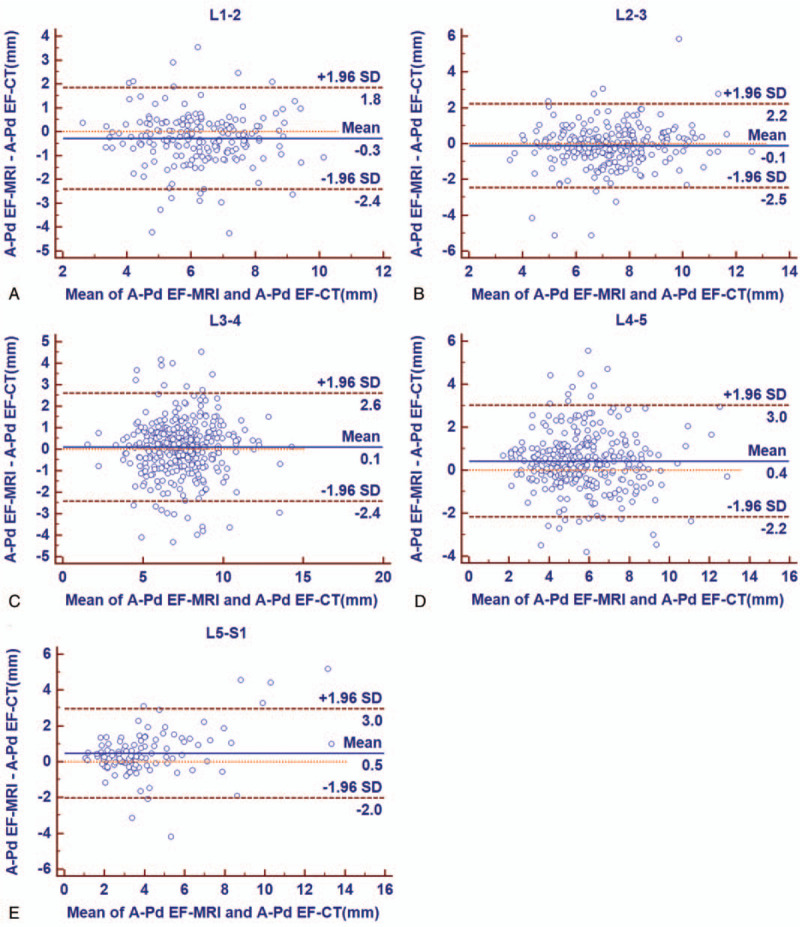
Bland–Altman plots showing satisfied agreement between CT and MRI measurements of fat diameter at L1–2, L2–3, L3–4, L4–5, and L5–1. The dashed line indicates the mean difference between the 2 methods (bias) and the dotted lines demonstrate 1.96 SD of the mean difference (limits of agreement). A-Pd EF = anteroposterior diameter of the epidural fat, CT = computed tomography, MRI = magnetic resonance imaging.

Bland–Altman plots showed a satisfied agreement between CT and MRI measurements of amounts of EF at all 5 disc levels (Fig. 6). From L1–2 to L5–S1, the mean difference of A-Pd EF using the 2 methods was −0.3, −0.1, 0.1, 0.4, and 0.5 mm, respectively; with the span of 95% limits of agreement ranged between −2.4 and 1.8 mm, −2.5and 2.2 mm, −2.4 and 2.6 mm, −2.2 and 3.0 mm, and −2.0 and 3.0 mm, respectively. Measurements of A-Pd DuS, Td DuS, CSA EF, and CSA DuS also showed significant correlation and satisfied agreement between CT and MRI (data not shown).

Of all levels available, A-Pd EF over 7 mm^[15]^ were found in 37.5% at L1–2, 60.8% at L2–3, 58.9% at L3–4, 20.6% at L4–5, and 5.6% at L5–S1 on CT images; and 27.6% at L1–2, 51.9% at L2–3, 60.6% at L3–4, 25.9% at L4–5, and 5.6% at L5–S1 on MR images (Table 2). When considering all levels together, we found that 41.2% CT images and 40.1% MR images can be radiologically diagnosed as LEL. We also compared CT diagnosis specificity of LEL with MRI, high agreement was detected in all levels, with kappa value varying from 0.62 to 0.76, all *P* < .001 dependent on different levels. Our result also indicated that LEL is more commonly seen in disc level of L2–3 (144/237, 60.8% on CT and 123/237, 51.9% on MRI) and L3–4 (212/360, 58.9% on CT and 218/360, 60.6% on MRI) (Chi-squared test, *P* < .001).

**Table 2:**
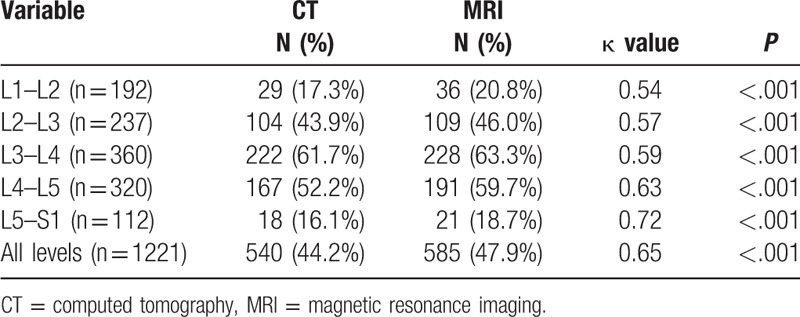
Level distribution and incidence of radiological spinal epidural lipomatosis in our study population.

### 3.3 Correlative factors of amounts of EF

A multiple linear regression model was established with the A-Pd EF as the dependent variable. In this model, gender, age, and body weight were significantly associated with amounts of EF (Table 3). Body weight factor was the strongest correlative factor in this model, with standardized coefficients being 0.208 on CT and 0.225 on MRI (both *P* < .001), while age factor showed lower coefficients which was 0.078 on CT and 0.067 on MRI. Surprisingly, our results indicated that female had a larger A-Pd EF than male with regression coefficients being 0.112 on CT and 0.169 on MRI. However, body height was not associated with amounts of EF in our present study.

**Table 3:**
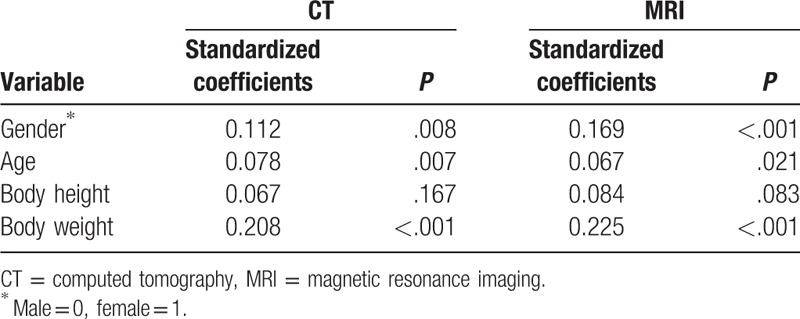
Multiple linear regression model for amounts of epidural fat.

## 4 Discussion

LEL is an uncommon entity, first described by Lee et al^[16]^ in 1975 in a patient receiving exogenous glucocorticoids status post renal transplantation. The EF acts as a contributing factor for spinal canal narrowing and makes the nerve root vulnerable to compression, and neurological symptoms occur due to a narrowing of the spinal canal caused by hypertrophy of the epidural adipose tissue. Conservative management with either weight loss or weaning from steroids can successfully reduce epidural adipose tissue leading to an improvement in symptoms.^[10,11,17]^ However, surgery is highly recommended and must be performed early by extensive decompression like multilevel laminectomies, fat debulking, and instrumented posterolateral fusion if conservative treatment failed. Considering surgical treatment as an end-stage selection and was associated with a high risk of postoperative morbidity,^[10,17]^ early detection of LEL via evaluating epidural adipose tissue is of great importance.

MRI is reported to be the investigation procedure of choice.^[18,19]^ The high contrast between adipose tissue and the DuS on T1-weighted images permits an accurate evaluation of EF. However, patients with heart pacemaker, metallic foreign body, and claustrophobia are not suitable for MRI. CT is an alternative choice when symptomatic patients with certain MRI contraindications need imaging evaluation. Few contraindications of CT scan have been reported except in pregnant females or using intravenous contrast medium. However, specificity and reproducibility of CT scan in EF assessment is still lacking in the literature. Our present study confirmed that epidural adipose tissue was distinguishable from periphery tissues on CT image by comparing HU values. Measurement of other adipose tissues in CT scan has been studied in animal models and clinical practices.^[20]^ Ross et al^[21]^ found no significant differences in visceral adipose tissue volume per slice when comparing 1.5 T MRI and CT in rats. Yoshizumi et al^[22]^ developed a practical, standardized technique for determining the abdominal fat area at CT. Klopfenstein et al^[23]^ even considered CT scan as a “gold standard" imaging modality for measurement of visceral adipose tissue area. In our present study, 5 major tissues that constitutes or surrounds spinal canal was identified by naked eyes and then marked; results showed significant differences of HU values among each type of tissue (Fig. 3), indicating that HU values of these tissues are obviously different that can be distinguished by naked eyes.

A series of studies has been published about the comparison of adipose tissue features between CT and MRI.^[23–25]^ However, the morphological features measurements of EF tissue have not been compared yet. In this study, the morphological features of EF from L1–2 through L5–S1 that were discernable on MRI and CT scan were evaluated and compared. No significant difference of A-Pd EF, A-Pd DuS, Td DuS, CSA EF, or CSA DuS between CT scan and MRI was found (Fig. 4). Close correlation and good agreement were identified between measurements of A-Pd EF on CT and MRI. These results indicated that CT scan has a similar capacity to detect adipose tissue when comparing to MRI.

Incidence of spinal level involvement was still controversial. Robertson et al^[15]^ reported idiopathic SEL seems to occur with equal frequency between the thoracic and lumbar spine. Fogel et al^[9]^ reported SEL often affects the thoracic (45.8%) and lumbosacral (43.6%) spine, and 10% of the patients may present with involvement of both, sparing the cervical spine. Ishikawa et al^[6]^ found the main lesion of SEL was located more frequently in lumbar spine than in the thoracic spine. As for lumbar spine, Ferlic et al^[5]^ concluded that SEL affected more frequently L5–S1 segment. However, our results indicated radiological SEL was more likely involved in L2–3 and L3–4 segments based on wider study population.

Individuals with A-Pd EF more than 7 mm were defined as radiological LEL, according to previous report.^[15]^ In general, application of CT scan in diagnosis of LEL in this study demonstrated great agreement with MRI, as κ = 0.68, *P* < .001(Table 2), this strongly indicated CT scan is an effective and specific tool in LEL evaluation.

In our present study, 41.2% levels on CT and 40.1 levels on MRI (Table 2) was radiologically diagnosed as LEL and was mostly seen in patients with higher body weight. This result was in consistence with previous reports.^[5,26,27]^ Male was reported to consist of 75% to 88% LEL patients in a few previously studies ^[7,15,28,29]^; however, surprisingly, our results indicated that female tended to have larger amounts of EF (Table 3). Furthermore, our results also indicated age was seemly associated with overgrowth of EF (Table 3).

Our present study suffers from a few limitations. First, the measurements of EF tissue were performed on CT or MR images typically based on a preselected sagittal angle of lumbar by technician, which were unable to be set as perfectly same between 2 methods. Second, only fat tissue that in the dorsal region of spinal canal, other than that of ventral and lateral region, was measured since it was reported to be the main component in the pathogenesis of thecal sac compression. Third, sample numbers varied apparently among different disc levels, especially in L5–S1 level with only 112 patients included due to special anatomic structure.

## 5 Conclusions

In conclusion, we demonstrated CT scan as a satisfied assessment method in detecting epidural adipose tissue significantly correlated with MRI. With its lower financial burden, shorter scan period, and fewer contraindications, CT scan could be considered as an optimistic alternative under some circumstances. Furthermore, we found that LEL is much more commonly seen at L2–3 and L3–4. High body weight, older age, and female gender are associated with overgrowth of EF.

## Author contributions

**Conceptualization:** Ziang Hu, Lili Wang.

**Data curation:** Yilei Chen, Zhaozhi Li, Xing Zhao.

**Formal analysis:** Shunwu Fan.

**Investigation:** Yilei Chen, Zhaozhi Li, Lijiang Song.

**Methodology:** Lijiang Song.

**Software:** Ziang Hu, Shunwu Fan, Xing Zhao.

**Supervision:** Lili Wang.

**Validation:** Ziang Hu, Shunwu Fan.

**Writing – original draft:** Yilei Chen, Ziang Hu, Lili Wang.

**Writing – review & editing:** Ziang Hu, Lili Wang.

## Correction

The funding information in the footnote did not appear when the article originally published and has now been added.
